# Gender differences in hospital admissions for major cardiovascular events and procedures in people with and without diabetes in England: a nationwide study 2004–2014

**DOI:** 10.1186/s12933-017-0580-0

**Published:** 2017-08-10

**Authors:** Anthony A. Laverty, Alex Bottle, Sung-Hee Kim, Bhakti Visani, Azeem Majeed, Christopher Millett, Eszter P. Vamos

**Affiliations:** Public Health Policy Evaluation Unit, School of Public Health, Imperial College London, London, W6 8RP UK

**Keywords:** Cardiovascular, Diabetes, Gender differences, Hospital admissions, Acute myocardial infarction, Stroke, Coronary revascularisation

## Abstract

**Background:**

Secondary prevention of cardiovascular disease (CVD) has improved immensely during the past decade but controversies persist on cardiovascular benefits among women with diabetes. We investigated 11-year trends in hospital admission rates for acute myocardial infarction (AMI), stroke, percutaneous coronary intervention (PCI), and coronary artery bypass graft (CABG) in people with and without diabetes by gender in England.

**Methods:**

We identified all hospital admissions for cardiovascular disease causes among people aged 17 years and above between 2004 and 2014 in England. We calculated diabetes-specific and non-diabetes-specific rates for study outcomes by gender. To assess temporal changes, we fitted negative binomial regression models.

**Results:**

Diabetes-related admission rates remained unchanged for AMI (incidence rate ratio (IRR) 0.99 [95% CI 0.98–1.01]), increased for stroke by 2% (1.02 [1.01–1.03]) and PCI by 3% (1.03 [1.01–1.04]) and declined for CABG by 3% (0.97 [0.96–0.98]) annually. Trends did not differ significantly by diabetes status. Women with diabetes had significantly lower rates of AMI (IRR 0.46 [95% CI 0.40–0.53]) and stroke (0.73 [0.63–0.84]) compared with men with diabetes. However, gender differences in admission rates for AMI attenuated in diabetes compared with the non-diabetic group. While diabetes tripled admission rates for AMI in men (IRR 3.15 [95% CI 2.72–3.64]), it increased it by over fourfold among women (4.27 [3.78–4.93]). Furthermore, while the presence of diabetes was associated with a threefold increased rates for PCI and fivefold increased rates for CABG (IRR 3.14 [2.83–3.48] and 5.01 [4.59–5.05], respectively) in men, among women diabetes was associated with a 4.4-fold increased admission rates for PCI and 6.2-fold increased rates for CABG (4.37 [3.93–4.85] and 6.24 [5.66–6.88], respectively). Proportional changes in rates were similar in men and women for all study outcomes, leaving the relative risk of admissions unchanged.

**Conclusions:**

Diabetes still confers a greater increase in risk of hospital admission for AMI in women relative to men. However, the absolute risk remains higher in men. These results call for intensified CVD risk factor management among people with diabetes, consideration of gender-specific treatment targets and treatment intensity to be aligned with levels of CVD risk.

## Background

Although many developed countries have documented substantial reductions in the incidence of cardiovascular disease (CVD) in diabetes in recent decades, one half of patients with diabetes die prematurely from a CVD cause, and over a quarter of all hospital admissions for CVD are diabetes related [1, 2]. In the general population, even in high CVD risk groups, the risk of fatal and non-fatal CVD events is over 20% lower among women, and women develop CVD events 5–10 years later in life on average than men [3, 4]. However, this pattern changes in diabetes [5]. While diabetes has been reported to double the risk of coronary heart disease among men with diabetes, it triples it among women with diabetes [6]. The risk of death following an acute myocardial infarction (AMI) is substantially higher in women compared with men with diabetes [7]. Furthermore, a pooled analysis estimated a 27% excess risk of stroke among women with diabetes compared with male counterparts [8]. In the presence of diabetes, major cardiovascular events have been estimated to present 20–30 years earlier in women and 15–20 years earlier in men compared with individuals free of diabetes [9]. Hence, among women, diabetes confers a greater risk of CVD compared with men to the extent that it eliminates, and to some degree reverses the ‘gender protection’ shown in women without diabetes [10].

The underlying mechanisms of the greater adverse impact of diabetes on CVD outcomes among women are not fully understood. Contributory factors may include gender differences in biological factors, risk factor profile and management of diabetes and its complications [5]. While women without diabetes have a more favourable CVD risk profile compared with men, this association alters in diabetes. Women have shown to be subject to more pronounced changes in a range of traditional and novel cardiovascular risk factors while transiting from a non-diabetic to a diabetic state [11]. As an example, men develop diabetes at lower levels of mean body mass index compared with women [12]. Therefore, women newly diagnosed with diabetes may already have a more adverse risk profile compared to men. These differences may be augmented by the under utilisation of evidence-based preventive and therapeutic interventions in women with diabetes that may, at least partly, follow from the underestimation of patient risk [13, 14].

Although secondary prevention has become more equitable between men and women with diabetes in England, women are still less likely than men to achieve treatment targets [15]. However, no previous studies have quantified to what extent substantial investments in secondary prevention translated into changes in major CVD events at a national level in England during the past decade, and whether men and women benefitted equally from CVD reductions. National clinical guidelines on the management of diabetes and quality improvement initiatives in primary care do not consider potential excess CVD risk among women with diabetes [1, 16]. The lack of recent epidemiological studies on this topic may have resulted in the notion that major CVD outcomes are equal in men and women or follow a similar pattern to that seen in the general population. In light of considerable targeted efforts to improve comprehensive secondary prevention in diabetes, potential gender inequalities in hard clinical outcomes have major policy implications.

Therefore, to address this gap in knowledge, our nationwide study aimed to assess trends in hospital admissions for pre-defined CVD outcomes in males and females with and without diabetes including AMI, stroke, percutaneous coronary intervention (PCI), and coronary artery bypass grafting (CABG), and associated inpatient mortality between 2004–2005 and 2014–2015 in England. We also assessed changes in the relative risk of study outcomes in women relative to men with and without diabetes during the study period.

## Methods

Hospital Episode Statistics (HES) is an administrative dataset that covers data on all inpatient hospital activity and day case admissions to National Health Service (NHS) hospitals in England, including private patients treated in NHS hospitals. We used HES data between 2004–2005 and 2014–2015 for all NHS hospital trusts in England. For each hospital admission, we extracted data on patient demographics (age and gender), length of hospital stay (LOS; inpatient days are calculated by subtracting the day of admission from the day of discharge), inpatient mortality, principal diagnosis on admission and secondary diagnostic codes (up to 19) using 10th revision of the International Statistical Classification of Diseases and Related Health Problems (ICD-10) codes. Procedures were identified using Office of Population Censuses and Surveys’ Classification of Surgical Operations (OPCS4) codes, that records up to 12 procedures.

We identified cardiovascular complications as the principal diagnosis on admission, including AMI (ICD-10 I21 and I22) and stroke (ICD-10 I60–I64). Cardiovascular procedures were identified using procedure codes for PCI (OPCS4 K49, K50, and K75) or CABG (OPCS4 K40–K46) in any procedure field. Diabetes status was identified based on type 1 or type 2 diabetes (ICD-10 codes E10 and E11) in any diagnostic field. Only the first admission was counted for patients with repeated admissions for the same cause during the same year. In total, we identified 754,500 hospital admissions for AMI (476,612 in men and 277,888 in women), 871,331 admissions for stroke (421,757 in men and 449,574 in women), 712,853 admissions for PCI (525,927 in men and 186,926 in women) and 233,882 admissions for CABG (184,919 in men and 48,963 in women) during the study period.

To calculate diabetes-specific admission rates, we used data from the Quality Management and Analysis System (QMAS) on the number of people with diabetes aged 17 years and above in England for each study year as denominator [17]. QMAS is the financial database for the Quality and Outcomes Framework (QOF), a national pay-for-performance scheme introduced in 2004 in UK primary care. QOF rewards general practices for delivering high quality care and aims to standardise improvements in the provision of primary care services across a range of areas, including the management of chronic conditions. Under QOF, general practices are financially incentivised to detect and record all diabetes cases among their practice populations, as practice payments are weighted by disease prevalence. Data in QMAS includes diabetes counts for people aged ≥17 years for virtually all (>99%) general practices in England. QMAS data do not record age and gender-specific information on diabetes. Therefore, we obtained data on the age and sex distribution of people with diabetes in England from the Health Survey for England (HSE) [18]. The prevalence of diabetes by age group and gender were missing in HSE datasets in years 2004, 2005, 2007 and 2008. Similar to previous studies, for the missing years, we used HSE data from 2006 (the mid-term of this 5-year period), allowing us to calculate age-and gender-specific admission rates for each study year [19, 20]. Diabetes-specific admission rates were calculated by gender and three separate age-bands (17–44, 45–64 and ≥65 years) using the total number of patients with diabetes in each gender- and age- band as denominator.

The admission rate of CVD events in people without diabetes for each year was calculated by deducting the number of people with diabetes from each corresponding age and gender stratum of the resident population, extracted from Office of National Statistics [21]. Therefore, the denominator for people without diabetes only included the number of people without diabetes.

The admission rates were directly age- and gender standardised using the population structure in the 1st year of study period (2004–2005) as the reference population. Rates were expressed per 100,000 people with or without diabetes. Inpatient mortality rates were calculated for people with and without diabetes.

Group differences between populations in 2004 and 2014 were tested using Chi square test for categorical variables and Student’s t test or Wilcoxon rank sum test for continuous variables, as appropriate. Female-to-male ratio for each study outcome was calculated as the ratio of the number of women admitted divided by the number of men admitted to hospital. Due to evidence on overdispersion, we fitted negative binomial regression models separately for patients with and without diabetes using age, gender and study year as independent variables. Interactions between diabetes versus year and gender versus year were tested for all outcomes. In a second set of models including the entire study population (people with and without diabetes), the relative risk of being admitted for CVD events and inpatient mortality were estimated comparing people with and with diabetes by gender (using men as reference) and comparing men and women by diabetes status (using people without diabetes as reference). Statistical analyses were performed using Stata version 14.0.

## Results

In people with diabetes, there was an increase in the absolute number of admissions for all study outcomes in both men and women between 2004 and 2014 in England. The baseline characteristics of people admitted for CVD by diabetes status and gender are shown in Table 1. The largest increase was evident for PCI, as the number of admissions more than doubled in both men and women during the study period. By contrast, in patients without diabetes, a decrease in the number of patients admitted for AMI and CABG was shown, and admissions for stroke and PCI increased moderately.

**Table 1:** Characteristics of people affected by cardiovascular disease by diabetes status and gender in England between 2004–2005 and 2014–2015

Outcome	Diabetes						No diabetes					
	Women			Men			Women			Men		
	2004–2005	2014–2015	P	2004–2005	2014–2015	P	2004–2005	2014–2015	P	2004–2005	2014–2015	P
AMI												
Number of events	4848	5982		7641	10,843		22,811	18,735		38,305	35,083	
% of males	–	–		61.2	64.5	<0.001^a^	–	–		62.7	65.2	<0.001^a^
Female-to-male ratio (95% CI)	–	–		0.63 (0.62–0.64)	0.55 (0.54–0.56)	<0.001^a^	–	–		0.59 (0.59–0.6)	0.53 (0.53–0.54)	<0.001^a^
Age, year, mean (SD)	74.9 (11.1)	73.7 (12.7)	<0.001^b^	69.3 (11.8)	69.4 (12.5)	0.727	76.2 (12.3)	75.2 (14.0)	0.001^b^	67.1 (13.7)	66.3 (14.1)	0.001^b^
Age groups, N (%) (years)												
17–44	72 (1.5)	121 (2.0)	<0.001^a^	211 (2.8)	292 (2.7)	<0.001^a^	406 (1.8)	437 (2.3)	<0.001^a^	2198 (5.7)	1986 (5.7)	<0.001^a^
45–64	709 (14.6)	1270 (21.2)		2203 (28.8)	3434 (31.7)		3448 (15.1)	3854 (20.6)		13,726 (35.8)	14,127 (40.3)	
≥65	4067 (83.9)	4591 (76.8)		5227 (68.4)	7117 (65.6)		18,957 (83.1)	14,444 (77.1)		22,381 (58.5)	18,970 (54.0)	
Median LOS, days (IQR)	5 (8–14)	3 (5–10)	<0.001^d^	5 (7–12)	3 (4–8)	<0.001^d^	5 (7–12)	2 (4–8)	<0.001^d^	4 (6–10)	2 (3–6)	<0.001^d^
Number of deaths (%)	881 (18.2)	676 (11.3)	<0.001^a^	1031 (13.5)	954 (8.8)	<0.001^a^	4417 (19.4)	1982 (10.6)	<0.001^a^	4453 (11.6)	2347 (6.7)	<0.001^a^
Stroke												
Number of events	4702	7678		4959	9600		34,079	34,947		29,005	33,247	
% of males	–	–		51.3	55.6	<0.001^a^	–	–		46.0	48.7	<0.001^a^
Female-to-male ratio (95% CI)	–	–		0.95 (0.94–0.95)	0.80 (0.80–0.81)	<0.001^a^	–	–		1.17 (1.17–1.2)	1.05 (1.04–1.05)	<0.001^a^
Age, year, mean (SD)	76.2 (11.1)	76.8 (11.9)	0.006^b^	72.0 (11.1)	72. 5 (11.6)	0.002^b^	77.1 (13. 4)	76.5 (14. 6)	<0.001^b^	71.4 (13.6)	71.1 (14.4)	0.002^b^
Age groups, N (%) (years)												
17–44	58 (1.2)	99 (1.3)	0.036^a^	82 (1.6)	176 (1.8)	0.405^a^	1116 (3.3)	1288 (3.7)	<0.001^a^	1353 (4.7)	1628 (4.9)	<0.001^a^
45–64	589 (12.5)	1086 (14.1)		1038 (20.9)	2081 (21.7)		4147 (12.2)	5181 (14.8)		6478 (22.3)	8151 (24.5)	
≥65	4055 (86.2)	6493 (84.6)		3839 (77.4)	7343 (76.5)		28,816 (84.6)	28,478 (81.5)		21,174 (73.0)	23,468 70.6)	
Median LOS, days (IQR)	6 (15–33)	3 (7–20)	<0.001^d^	5 (11–28)	2 (6–16)	<0.001^ε^	5 (12–29)	2 (7–19)	<0.001^d^	4 (10–24)	2 (5–14)	<0.001^d^
Number of deaths (%)	1485 (31.6)	1438 (18.7)	<0.001^a^	1191 (24.0)	1410 (14.7)	<0.001^a^	11,614 (34.1)	7005 (20.4)	<0.001^a^	7476 (19.5)	4812 (13.7)	<0.001^a^
CABG												
Number of events	1028	1111		3531	4275		4029	2649		15,165	11,005	
% of males	–	–		77.5	79.4	0.02^a^	–	–		79.0	80.6	<0.001^a^
Female-to-male ratio (95% CI)	–	–		0.29 (0.27–0.3)	0.26 (0.25–0.27)	0.02^a^	–	–		0.26 (0.25–0.27)	0.24 (0.23–0.25)	<0.001^a^
Age, year mean (SD)	67.5 (8.5)	68.1 (9.8)	0.105^b^	65.1 (8.7)	66.7 (9.5)	<0.001^b^	69.2 (9.1)	70.3 (10.1)	<0.001^b^	65.4 (9.4)	67.1 (10.2)	<0.001^b^
Age groups, N (%) (years)												
17–44	13 (1.3)	16 (1.4)	0.940^a^	57 (1.6)	64 (1.5)	0.004^a^	63 (1.6)	40 (1.5)	0.610^a^	317 (2.1)	199 (1.8)	<0.001^a^
45–64	322 (31.3)	348 (31.3)		1460 (41.3)	1612 (37.7)		966 (24.0)	608 (30.0)		6217 (41.0)	3916 (35.6)	
≥65	693 (67.4)	747 (67.2)		2014 (57.0)	2599 (60.8)		3000 (74.5)	2001 (75.5)		8631 (56.9)	6890 (62.6)	
Median LOS, days (IQR)	8 (11–20)	8 (11–18)	0.086^d^	7 (9–16)	7 (9–14)	<0.001^d^	7 (10–16)	7 (10–17)	0.672^c^	7 (8–13)	6 (9–14)	0.041^c^
Number of deaths (%)	53 (5.1)	30 (2.7)	0.003^a^	102 (2.8)	69 (1.6)	<0.001^a^	219 (5.4)	131 (4.9)	0.379^a^	412 (2.7)	256 (2.3)	0.054^a^
PCI												
Number of events	2238	4576		5106	11,862		11,190	14,764		31,386	43,178	
% of males	–	–		69.5	72.2	<0.001^a^	–	–		73.7	74.5	0.004^a^
Female-to-male ratio (95% CI)	–	–		0.44 (0.42–0.45)	0.38 (0.37–0.39)	<0.001^a^	–	–		0.36 (0.35–0.37)	0.34 (0.33–0.34)	0.004^a^
Age, year, mean (SD)	65.3 (10.7)	67.9 (11.8)	<0.001^b^	63.2 (10.1)	65.5 (11.2)	<0.001^b^	66.4 (10.5)	68.9 (12.2)	<0.001^b^	61.7 (10.9)	63.4 (12.2)	<0.001^b^
Age groups, N (%) (years)												
17–44	91 (4.1)	152 (3.3)	<0.001^a^	217 (4.2)	402 (3.4)	<0.001^a^	348 (3.1)	416 (2.8)	<0.001^a^	1996 (6.4)	2438 (5.6)	<0.001^a^
45–64	850 (38.0)	1513 (33.1)		2404 (47.1)	4982 (42.0)		4066 (36.3)	4585 (31.1)		16,445 (52.4)	20,416 (47.2)	
≥65	1297 (58.0)	2911 (63.6)		2485 (48.7)	6478 (54.6)		6776 (60.6)	9763 (66.1)		12,945 (41.2)	20,324 (47.1)	
Median LOS, days (IQR)	1 (2–6)	1 (2–5)	0.013^d^	1 (2–5)	1 (2–4)	<0.001^d^	1 (2–4)	1 (2–4)	<0.001^d^	1 (2–4)	1 (2–3)	<0.001^d^
Number of deaths (%)	45 (2.0)	146 (3.2)	0.006^a^	65 (1.3)	253 (2.1)	<0.001^a^	142 (1.3)	426 (2.9)	<0.001^a^	282 (0.9)	851 (2.0)	<0.001^a^

*AMI* acute myocardial infarction, *CABG* coronary artery bypass grafting, *PCI* percutaneous coronary intervention, *LOS* length of hospital stay, *IQR* interquartile range, *CI* confidence interval, *SD* standard deviation

^a^Chi square test

^b^Student’s t test

^c^Wilcoxon test—2004 figure lower

^d^Wilcoxon test—2004 figure higher

There was a considerable male excess in both diabetic and non-diabetic groups for all outcomes except for stroke (Table 1). The greatest male excess occurred in admissions for cardiovascular interventions in both groups. The female-to-male ratio significantly decreased for all outcomes during the study period representing an increasing male predominance. As expected, most admissions for CVD events and procedures occurred among people over 65 years of age. Notably, women admitted for AMI were 5 years older on average in the diabetes group and 9 years older in the non-diabetic group compared with men in corresponding groups. Length of hospital stay (LOS) significantly reduced for AMI, stroke and PCI admissions in the diabetes and non-diabetes groups in both men and women between 2004 and 2014. For CABG, while a reduction occurred in LOS in all groups, it remained unchanged in women without diabetes.

### Changes in admission rates

**Fig. 1:**
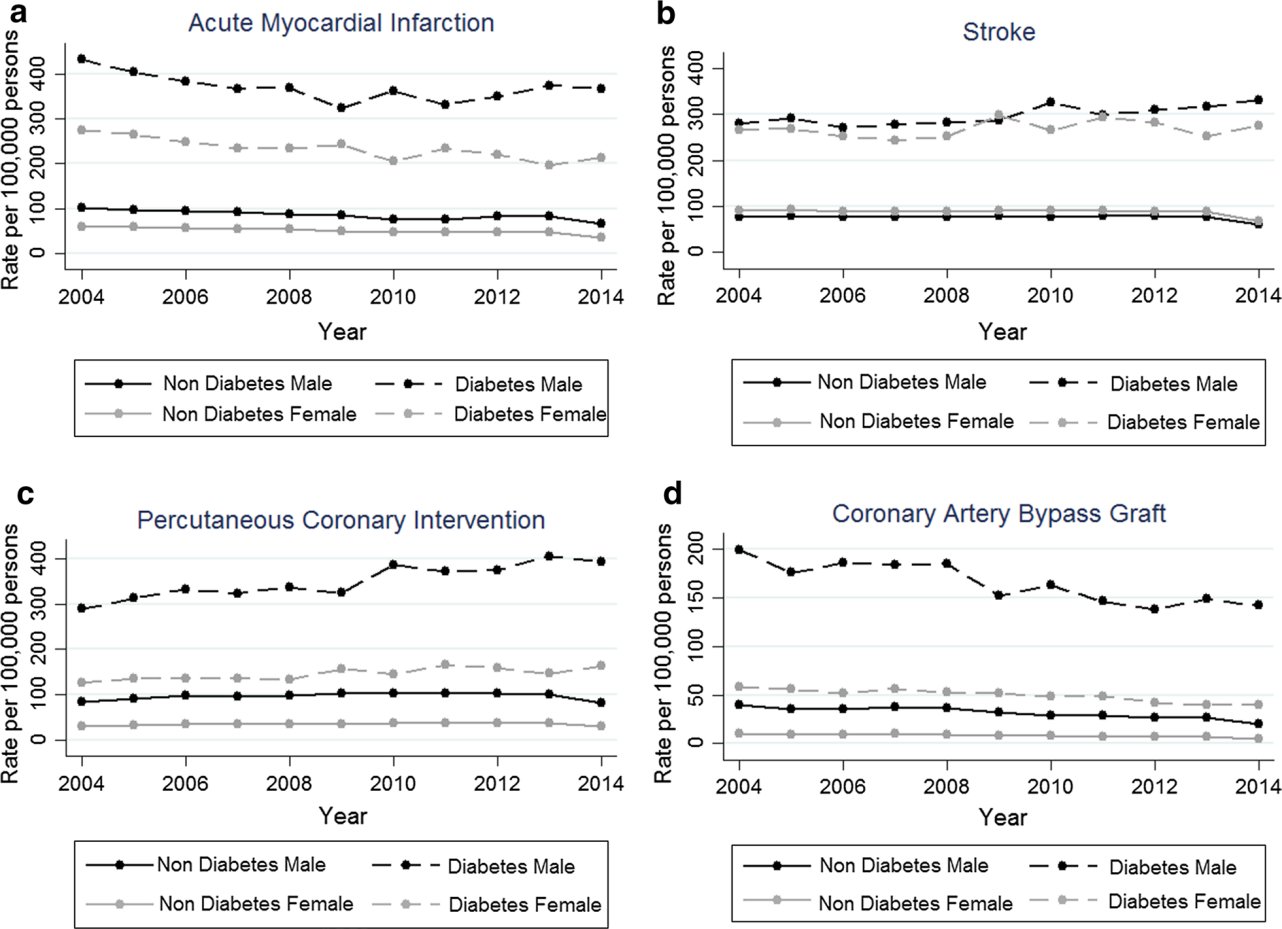
Age- and gender-standardized rates of admissions by year and gender. **a** Acute myocardial infarction; **b** stroke; **c** percutaneous coronary intervention; **d** coronary artery bypass grafting. Rates are expressed as 100,000 people with diabetes (diabetes group) or 100,000 people without diabetes (non diabetes group)

Figure 1 shows the age- and gender-standardised rates for all study outcomes over the 11-year study period in people with and without diabetes. Men had higher absolute rates in both groups for all outcomes except for stroke in the non-diabetes group (Fig. 1). Although age- and gender-standardised admission rates for AMI showed reductions in the diabetes group, these temporal changes did not reach statistical significance in regression models after adjustment for study covariates (Fig. 1; Table 2). Diabetes-related admission rates for stroke and PCI increased significantly with rate ratios of 1.02 (95% CI 1.01–1.03) and 1.03 (10.01–1.04), representing an annual an increase of 2 and 3%, respectively (Fig. 1; Table 2). In people without diabetes, admissions for AMI declined statistically significantly (IRR (95% CI) 0.98 (0.95–0.99)) and remained stable for stroke and PCI. Both groups experienced significant reductions in CABG rates.

**Table 2:** Rate ratio of hospital admissions for CVD events and interventions in people with and without diabetes between 2004 and 2014 in England

Outcome	Changes in rates									
	Hospital admissions, rate ratio (95% CI)								In-patient mortality, rate ratio (95% CI)	
	Total population			Male		Female		P value gender versus year^c^	Total population	
	Rate ratio^a^	P	P value diabetes versus year^b^	Rate ratio^a^	P	Rate ratio^a^	P		Rate ratio^a^	P
AMI										
Diabetes	0.99 (0.98–1.01)	0.587	0.311	1.00 (0.98–1.01)	0.626	0.99 (0.98–1.00)	0.096	0.470	0.95 (0.93–0.97)	<0.001
No diabetes	0.97 (0.95–0.99)	0.039		0.97 (0.96–0.98)	<0.001	0.98 (0.97–0.99)	<0.001	0.992	0.96 (0.94–0.98)	<0.001
Stroke										
Diabetes	1.02 (1.01–1.03)	0.005	0.309	1.03 (1.01–1.04)	<0.001	1.01 (1.00–1.02)	0.062	0.178	0.95 (0.93–0.96)	<0.001
No diabetes	1.00 (0.99–1.01)	0.993		1.00 (0.99–1.01)	0.930	0.99 (0.99–1.01)	0.791	0.340	0.94 (0.93–0.96)	<0.001
PCI										
Diabetes	1.03 (1.01–1.04)	<0.001	0.109	1.03 (1.02–1.04)	<0.001	1.02 (1.01–1.03)	0.002	0.200	1.07 (1.05–1.09)	<0.001
No diabetes	1.01 (0.97–1.03)	0.586		1.01 (1.00–1.02)	0.131	1.00 (0.99–1.01)	0.497	0.914	1.10 (1.07–1.12)	<0.001
CABG										
Diabetes	0.97 (0.96–0.98)	<0.001	0.008	0.97 (0.96–0.98)	<0.001	0.96 (0.95–0.97)	<0.001	0.552	0.96 (0.94–0.98)	<0.001
No diabetes	0.94 (0.93–0.95)	<0.001		0.95 (0.94–0.95)	<0.001	0.94 (0.93–0.95)	<0.001	0.719	0.98 (0.97–0.99)	<0.001

*AMI* acute myocardial infarction, *CABG* coronary artery bypass grafting, *PCI* percutaneous coronary intervention, *CI* confidence interval

^a^Rate ratios are obtained from negative binomial regression models adjusted for age, gender and study year

^b^P value is based on interaction between diabetes status and study year from negative binomial regression models

^c^P value is based on interaction between gender and study year from negative binomial regression models

During the 11-year study period, people with and without diabetes experienced similar proportional changes in admission rates for all study outcomes (Table 2). There were no statistically significant differences in trends between women and men in either group leaving the relative risk of events unchanged during the study period (Table 2).

### Relative risk of CVD

Table 3 shows the Ratio Ratios for study outcomes obtained from negative binomial regression models comparing people with and with diabetes by gender (using men as reference) and comparing men and women by diabetes status (using people without diabetes as reference). Compared with males without diabetes, women without diabetes had approximately three-times lower admission rates for AMI, 14% lower for stroke, 3.7-times lower for PCI and five-times lower for CABG, after adjustment for study covariates.

**Table 3:** Risk of major cardiovascular events and procedures in males and females with and without diabetes between 2004–2005 and 2014–2015 in England

Rate ratio (95% CI)								
	No diabetes^a^		Diabetes^b^		Male^a^		Female^c^	
	Male (reference)	Female	Male (reference)	Female	No diabetes (reference)	Diabetes	No diabetes (reference)	Diabetes
Admissions								
AMI	1.00	0.33 (0.29–0.39)^d^	1.00	0.46 (0.40–0.53)^d^	1.00	3.15 (2.72–3.64)^d^	1.00	4.27 (3.78–4.93)^d^
Stroke	1.00	0.86 (0.75–0.99)^e^	1.00	0.73 (0.63–0.84)^d^	1.00	2.67 (2.31–3.09)^d^	1.00	2.29 (1.94–2.60)^d^
PCI	1.00	0.27 (0.24–0.30)^d^	1.00	0.37 (0.34–0.42)^d^	1.00	3.14 (2.83–3.48)^d^	1.00	4.37 (3.93–4.85)^d^
CABG	1.00	0.22 (0.20–0.24)^d^	1.00	0.28 (0.25–0.30)^d^	1.00	5.01 (4.59–5.05)^d^	1.00	6.24 (5.66–6.88)^d^
In-patient mortality								
AMI	1.00	0.59 (0.51–0.68)^d^	1.00	0.59 (0.51–0.68)^d^	1.00	0.68 (0.59–0.79)^d^	1.00	1.17 (1.01–1.34)^f^
Stroke	1.00	0.99 (0.86–1.15)	1.00	0.89 (0.76–1.04)	1.00	0.69 (0.59–0.80)^d^	1.00	0.62 (0.52–0.71)^d^
PCI	1.00	0.47 (0.41–0.52)^d^	1.00	0.52 (0.45–0.60)^d^	1.00	1.18 (1.04–1.33)^e^	1.00	1.32 (1.15–1.51)^d^
CABG	1.00	0.48 (0.43–0.51)^d^	1.00	0.51 (0.45–0.57)^d^	1.00	0.93 (0.85–1.02)	1.00	1.01 (0.91–1.12)

*AMI* acute myocardial infarction, *CABG* coronary artery bypass grafting, *PCI* percutaneous coronary intervention, *CI* confidence interval

^a^Rate ratios are obtained from negative binomial regression models adjusted for age, sex, diabetes status and study year with men without diabetes as reference

^b^Rate ratios are obtained from negative binomial regression models adjusted for age, sex, diabetes status and study year with men with diabetes as reference

^c^Rate ratios are obtained from negative binomial regression models adjusted for age, sex, diabetes status and study year with women without diabetes as reference

^d^P < 0.001

^e^P < 0.01

^f^P < 0.05

By contrast, in the presence of diabetes, women had approximately half of the admission rates for AMI (IRR 95% CI 0.46 (0.40–0.53)) relative to men with diabetes. Although women with diabetes were significantly less likely to undergo a PCI or CABG compared with men with diabetes, the rate ratios are higher than that seen when comparing men and women without diabetes (Table 3). These figures may represent a moderate attenuation of the female advantage seen in the no diabetes group for admissions for AMI and revascularisation procedures. Women with diabetes had 27% lower admission rates for stroke compared with men with diabetes (Table 3).

While the presence of diabetes in men tripled the risk of being admitted for AMI (IRR (95% CI) 3.15 (2.72–3.64)) and increased the risk of stroke admissions by 2.7-times (2.67 (2.31–3.09)) (i.e. men with diabetes vs. men without diabetes), in women diabetes was associated with a 4.3-fold increased risk of admission for AMI (IRR (95% CI) 4.27 (3.8–4.93), P < 0.001) and a 2.3-fold increased risk of stroke (2.29 (1.94–2.60), P < 0.001) (i.e. women with diabetes vs. women without diabetes). Similarly, while in men diabetes was associated with an approximately threefold increased rates of PCI and fivefold increased rates of CABG, among women diabetes was associated with fourfold increased PCI admission rates and 6.2-fold increased CABG rates (Table 3).

### Inpatient mortality rates

There was a statistically significant decline in inpatient mortality rates in people with and without diabetes between 2004 and 2014 for all study outcomes except for PCI (Tables 1, 2). The results of multivariate negative binomial regression models show that inpatient mortality rates are lower among women compared with men in both people with and without diabetes for all outcomes but stroke admissions (Table 3). Men with diabetes experienced lower rates of inpatient mortality related to AMI and stroke admissions than men without diabetes after adjustment for study co-variates. By contrast, women with diabetes had higher rates of inpatient mortality following AMI admissions and lower rates of inpatient mortality related to stroke admissions compared with females free of diabetes (Table 3).

## Discussion

Our data revealed diverse trends in hospital admissions for major CVD events and coronary revascularisation procedures in men and women with and without diabetes between 2004 and 2014 in England. Among people with diabetes, rates of hospital admissions for AMI remained unchanged, increased for stroke and PCI and declined for CABG over the 11-year study period. There were no statistically significant differences in trends between people with and without diabetes during the study period for any of the study outcomes except for CABG. Our analyses of national data showed that although the absolute admission rates were higher among men for all outcomes, diabetes among women was associated with a greater risk of hospital admissions for AMI and revascularisation procedures but not for stroke. While the presence of diabetes was associated with tripled admission rates for AMI in men, it increased rates by 4.3-fold among women. Similarly, while men with diabetes had 3-times higher admission rates for PCI and 5-times higher admission rates for CABG, among women the presence of diabetes was associated with 4.4-fold increased PCI and 6.2-fold increased CABG rates compared with women without diabetes. Men and women experienced similar proportional changes in admission rates for all study outcomes, leaving the relative risk of events unchanged during the study period. Women with and without diabetes experienced lower inpatient mortality rates than men for all admissions except for stroke.

### Temporal changes in cardiovascular morbidity related to diabetes

The past decade has seen immense advances in targeted and population-wide approaches to the prevention and management of CVD [22]. However, it is not known how these efforts have affected contemporary patterns of CVD morbidity in England, and earlier studies may not provide accurate reflections on current trends. Many developed countries have documented downward trends in acute CVD events and related mortality in people with diabetes [23–26]. Some studies reported larger reductions in AMI and stroke rates in diabetes and, therefore, narrowing differences in event rates between people with and without diabetes [23, 24]. Our results correspond with those studies that found similar temporal changes in CVD event rates people with and without diabetes [20, 25].

Our observations of unchanged hospital admission rates for AMI and increased stroke rates in diabetes are important as there have been considerable investments in England targeting the early detection and management of diabetes and its complications, and the optimisation of CVD risk factor control. Although it is difficult to make comparisons across studies due to differences in case definitions and observation periods, this finding differs from the results of reduced diabetes-related admissions for AMI and unchanged stroke rates found by our previous analyses over a shorter observation period, between 2004 and 2009 [20]. The Quality and Outcomes Framework (QOF), the world’s largest pay-for-performance scheme in primary care that was introduced in 2004 in England, resulted in improved electronic recording, advanced processes of care and better intermediate outcomes for diabetes [27]. However, it has been contested whether these improvements were due to pre-existing trends, and to what extent they translated into better clinical outcomes for patients (such as prevention of AMI and stroke) [28]. The recorded attenuation of improvements in intermediate outcomes after the early years of QOF (including blood pressure, cholesterol and glycated haemoglobin levels) may explain the levelling off of potential clinical benefits over a longer period of time [27, 28]. Furthermore, advances in clinical management may not have detectable impact on hard clinical outcomes due to other important influences such as changes in the makeup of people with diabetes, longer life expectancy of patients and the socio-economically patterned distribution of comorbidity across population subgroups [29]. The recorded fall in mean blood pressure, cholesterol, glycated haemoglobin levels and smoking prevalence may also be off-set by the increase in body mass index [30].

Increase in admissions among people with diabetes for stroke might reflect improving survival of patients following cardiac events and longer life expectancy rather than the insufficiency of stroke prevention [23]. The finding of increased volume of coronary revascularisations with downward trends in the use of CABG and a parallel increase in PCI corresponds with previous studies [31, 32]. These trends may reflect a substantial shift towards less invasive revascularisation procedures [31].

### Gender differences in the risk of cardiovascular morbidity associated with diabetes

Strong evidence suggests that diabetes confers a greater excess risk of cardiovascular morbidity and mortality in women compared with men [6–8]. While some, but not all, studies reported equal or higher absolute risk of cardiovascular disease in women with diabetes relative to men, studies have repeatedly documented a greater effect of diabetes on CVD outcomes among female compared with male patients [7, 9, 11]. The results of our study correspond with those that observed a greater absolute risk of cardiovascular outcomes among men compared with women with diabetes but found a higher relative risk of coronary heart disease related to diabetes in women compared with men [7, 9, 33]. While diabetes was associated with tripled admission rates for AMI in men, it conferred an over fourfold increase in rates among women. Somewhat lower figures were reported by a meta-analysis that found that the relative risk of incident CHD in people with diabetes relative to those without diabetes was 2.16 in men and 2.82 in women [6]. We are not aware of other nationwide studies investigating gender differences in hospital admissions for major CVD causes associated with diabetes over a longer period of time in England. A cohort study from the UK found a moderately higher risk of non-fatal AMI among women compared with men with Type 2 diabetes but only among younger people below the age of 60 years [34].

Women with diabetes in our study had slightly lower rates of stroke compared with men with diabetes after adjustment for age, and the presence of diabetes was not associated with greater admission rates for stroke in female compared with male patients. While diabetes conferred a 2.7-fold increased rates for stroke admissions in men, it increased rates by 2.3-fold among women. The similar excess risk for stroke among women and men corresponds with the results of some previous studies and has been suggested to follow from the reduced impact of diabetes on CVD risk with an increasing age [5, 9, 34].

Although our study does not provide explanations for the stronger association between diabetes and AMI among women relative to men, several potential mechanisms have been suggested by previous studies [5, 35]. These mechanisms include physiological, clinical and management factors related to CVD that have been suggested to differ by gender. Many studies observed a greater deterioration in a range of conventional and novel cardiovascular risk factors in women compared with men while progressing from a non-diabetic to a diabetic state [5, 11, 36]. These risk factors encompass inflammation, impaired fibrinolysis, coagulation, dyslipidemia, hypertension and endothelial dysfunction, amongst other metabolic and hormonal factors [11, 36–38]. Gender differences in the association between diabetes and CVD risk factors have been partly attributed to differences in central adiposity and insulin resistance [11, 12, 35, 37]. Women tend to have peripheral fat distribution by contrast to central obesity with visceral fat accumulation in men, resulting in improved insulin sensitivity even at greater levels of weight gain [11]. Women, therefore, need to undergo more profound changes in metabolic risk factors (including body mass index and insulin resistance) to develop diabetes and tend to present a more adverse CVD risk factor profile at diagnosis [12, 36].

It has been widely documented that the heavier risk factor burden among women is compounded by the suboptimal use of diagnostic procedures and evidence-based interventions, less aggressive treatment strategies and a lower attainment of clinical treatment targets during the course of diabetes [7, 35, 39]. These gender disparities have been repeatedly documented in England and worldwide and seem to persist over time [5, 15, 35, 40]. As an example, a large cohort study from England found that women with diabetes were less likely than men to have their glycated haemoglobin and cholesterol recorded, were less likely to be prescribed antiglycaemic and lipid-lowering medications and had poorer blood pressure, cholesterol and glycaemic control [15]. Some of these gender disparities may stem from the notion that women are ‘protected’ against CVD resulting in the underestimation of the risk of serious acute CVD events and long-term CVD risk, and may also affect women’s self-awareness of their own risk [35]. Gender differences in the clinical presentation of acute coronary syndrome may result in delayed or missed diagnoses [35]. Furthermore, some early reports suggesting that women may benefit less than men from certain revascularisation procedures and pharmacological treatments in CVD risk reduction that may have led to the underuse of preventative and therapeutic interventions [41].

Gender disparities in cardiovascular disease outcomes have important clinical and public health implications. Despite the repeated documentations of a more adverse impact of diabetes on CVD profile and higher relative risk of fatal and non-fatal CHD among women, CVD is still under-recognised and undertreated among women [35, 41]. Importantly, women with AMI have been reported to have more unfavourable outcomes following an AMI including higher in-hospital and 1-year mortality [35]. Although not specifically assessed by our study, previous analyses highlighted a greater relative risk of AMI and higher complication and mortality rates following AMI, specifically among younger women compared with similarly aged men [35, 41]. These acknowledged gender disparities could serve as potential targets for interventions. However, clinical guidelines and quality improvement initiatives do not specifically recognise the unique risk factor profile of women with diabetes [16]. Recognising this gap, the American Heart Association published a scientific statement and recommendations for earlier and more targeted interventions for the prevention of CVD among women [42].

### Strengths and limitations

To our knowledge, this is the first national study describing recent trends of hospital admissions for CVD outcomes in men and women by diabetes status England over the past decade. Our study covers the entire population of England and given that all study outcomes require hospital admission, our results are likely to provide an accurate reflection on the burden of CVD in people with diabetes. Furthermore, we had data on the number of people with diabetes in England for each study year, allowing the estimation of diabetes-specific event rates.

Limitations of our study include that we cannot directly evaluate miscoding, misdiagnosis and misclassification of diagnoses in the HES database. HES is an administrative database, and given that the reimbursement of NHS hospitals for each patient seen is directly determined by coding data, hospitals have a strong financial incentive to establish and maintain accurate coding practices. Routinely collected data are subject to regular national clinical coding audits and a systematic review of discharge coding evaluated its accuracy high for both diagnoses and procedures [43]. HES do not include data on the clinical, laboratory and lifestyle characteristics of individuals admitted to NHS hospitals in England. Therefore, we were unable to adjust our analyses for changes in cardiovascular risk factors of patients requiring hospital admission for CVD causes during the study period. However, analysing national hospital activity data in consecutive years provides an important and accurate reflection on temporal changes in the burden of major CVD events and procedures at a national level. We did not report Type 1 and Type 2 diabetes-specific events separately because national diabetes registers do not include information on diabetes type. We used data from Health Survey for England to ascertain the age and gender distribution of diabetes in England. For 2004, 2005 and 2007 and 2008 when age- and gender-specific data were not available we used data from 2006, the mid-term of this 5-year period.

## Conclusions

In conclusion, in this national study covering the entire population of England, we found unchanged admission rates for AMI and increased rates for stroke in people with diabetes between 2004 and 2014. The consistent upward trend in the number of diabetes-related admissions reflect the increasing absolute burden of CVD complications in diabetes. Although women with diabetes had lower absolute CVD event rates compared with men, diabetes conferred a larger relative increase in risk for hospital admissions for AMI in women compared with men. These observations call for aligning CVD risk with the intensity of preventive strategies in England and a more aggressive cardiovascular risk factor management among women.
